# Indications and Case Series of Intentional Replantation of Teeth 

**Published:** 2013-12-24

**Authors:** Saeed Asgary, Laleh Alim Marvasti, Alireza Kolahdouzan

**Affiliations:** a *Iranian Center for Endodontic Research, Research Institute of Dental sciences, Shahid Beheshti University of Medical Sciences, Tehran, Iran; *; b *Dental Research Center, Research Institute of Dental sciences, Shahid Beheshti University of Medical Sciences, Tehran; Iran *; c *Department of Endodontics, Dental School, Qazvin University of Medical Sciences, Qazvin, Iran*

**Keywords:** Biomaterial, Calcium Enriched Mixture, CEM Cement, Review Literature, Root Canal Therapy, Surgical Endodontics

## Abstract

This case series aims to comprehensively introduce intentional replantation with a focus on its indications and case selection in endodontics. In all represented cases, calcium enriched mixture (CEM) cement is used for root-end filling. This case series demonstrates twenty cases of IR and extraoral root-end resection and filling with CEM cement. All the selected teeth had a failed endodontic treatment and required surgical/nonsurgical endodontic (re)treatment or extraction. Subsequent to gentle tooth extraction, an appropriate root-end cavity was prepared and filled with CEM cement. Then the tooth was replanted; maximun procedure time was 15 min. A total of 18 cases (90%) were successful over a mean follow-up period of 15.5 months. It can be concluded that intentional replantation with careful case selection can have a high success rate over 2 years. Intentional replantation may be a suitable treatment option for both trained general practitioners and specialists provided that the extraction is simple and straightforward.

## Introduction

Intentional replantation (IR) is a surgical procedure that has often been regarded as the last treatment option [1]. It is less popular than implant and endodontic retreatment. This decade has shown a rising interest in IR with (bio)materials in several dental disciplines, including root-end biomaterials, and periodontal regenerators [2, 3]. 

In endodontics, IR involves the atraumatic extraction of the offending tooth, root-end resection/preparation/filling and reinsertion of the extracted tooth [4]. Recent case reports have demonstrated that with good case selection, IR can be a reliable and predictable procedure [5-7]; however, case studies do not have the same weight as randomized clinical trials. There are several key factors that must be considered when choosing a clinical option including: 1) patient factors and physical limitations; 2) endodontic and anatomic tooth factors; and 3) operator factors. Often, the thought of such a procedure appears far more fraught with complications than reality. *Operator factors* are the most significant as the practitioner should have the skill and confidence in atraumatic extractions [8]; and have knowledge and availability of dental biomaterials. IR is ideal when the operator desires superb access and visualisation of root apex and furcation or where [7, 9] the operator wishes to address both periapical and periradicular infection. 

*Tooth factors* include iatrogenic obstacles (such as crowns, posts or fractured instruments), complicated coronal endodontic retreatment [8], anatomical obstacles such as root canal obliteration, grossly overfilled canals, seemingly adequate orthograde endodontic treatment that has failed unresolved/unexpected pathosis and pain after conventional endodontic (re)treatment. Also IR is indicated when periradicular surgery is being considered but extensive bone removal is required; in cases of odontogenic maxillary sinusitis associated with an infected tooth, when all other endodontic treatments have failed, difficult access for periradicular surgery (close proximity of anatomical structures such as mental nerve), if root fracture is suspected but not definitively diagnosed and periodontally involved teeth [4, 6, 8, 10]. Other cases where unintentional extraction occurs or where there are dental anomalies [2, 8, 11]. 

Patient factors may also influence our decision to carry out IR including patients’ rejection of periradicular surgery; difficult intraoral access and visualisation [1, 7]; patients’ rejection of retreatment (*e.g.* access preparation through an expensive recently placed crown); and skeletal growth in young patients making implant treatment undesirable.

Many cases selected for IR are treated for several different reasons. IR may be more suitable for second molars which are likely to have fused/convergent roots, and for single rooted teeth [4]. However, each case and the operator’s ability must be assessed individually. For example, the presence of extensive apical bone loss around lower first molars will invariably allow easier extraction irrespective of the root divergence. IR may also impose fewer risks and cause less complications than periradicular surgery, *e.g.* nerve injury, or maxillary sinus access and complications [4]. 

Suitable case selection, the etiology of the affected tooth and the indication for IR will also have a remarkable influence on treatment prognosis. Experts recommend that the extra-oral time taken to restore the tooth will directly affect the prognosis of IR [1, 11]. The manipulation and damage to the periodontal ligament (PDL), cementum and associated cells should also be kept minimum, in order to keep the cells viable/intact [6, 12]. There are case studies with long term successful follow-ups (as long as 15 years) [11, 13]. IR of teeth that are hermetically sealed with a retro- or orthograde root-end filling is proved to be more successful. The biocompatibility of the filling material will also affect the healing process [14]. 

Calcium enriched mixture (CEM) cement was developed as an endodontic biomaterial, with good sealing ability [15], antibacterial effect [16], biocompatibility [16], and cementogenic effect [17]; with its indications being comparable to MTA,

Case studies on IR since 1999 are demonstrated in Table 1. In the present case series, we have focused on 20 cases of IR in endodontically failed teeth with CEM cement as root-end filling material.

## Methods and Materials

A total of 20 patients that were referred to private endodontic practice in Northern Tehran over a period of 2 years were selected for IR and consented to have IR treatment. The same endodontist conducted the treatment for all the 20 patients. Case selection was based on patient factors, and on extra/intra-oral examination. Before surgical intervention being considered, critical parameters such as root length/shape, amount of left tooth structure, amount of remaining bone/extent of osseous destruction, soft tissue attachment level, risk to adjacent teeth/restoration and patient’s oral hygiene were meticulously evaluated. Only those teeth/patients that fulfilled the suitable criteria for replantation were offered IR.

All possible treatment options were explained to the patient including *i)* tooth extraction with/without replacement, *ii)* retreatment and coronal restoration, *iii)* periradicular surgery, and *iv)* IR with root-end filling. The risks and benefits of each option were thoroughly explained and the patients signed a written informed consent. 

All teeth had previously received root canal therapy, except one. In all cases, medical histories revealed no contraindication to dental treatment. One patient was on warfarin and after consultation with cardiologist and after taking the INR test in the morning which was 2, treatment was conducted similar to other patients but in the afternoon.

Patients were given 400 mg of Ibuprofen (Darou Pakhsh, Tehran, Iran) preoperatively to prevent postoperative pain. A 0.2% Chlorhexidine rinse (Shahrdaru, Tehran, Iran) was also carried out to control the oral microflora. 

The clinical procedures for IR were as follows: after achieving complete local anesthesia (2% lidocaine with 1:80000 adrenalin; Darou Pakhsh, Tehran, Iran), the teeth were intentionally and gently extracted by means of a suitable periotome. The PDL and root surface area was left untouched. A skilled dental assistant hydrated the teeth with constant saline irrigation. 

**Figure 1:**
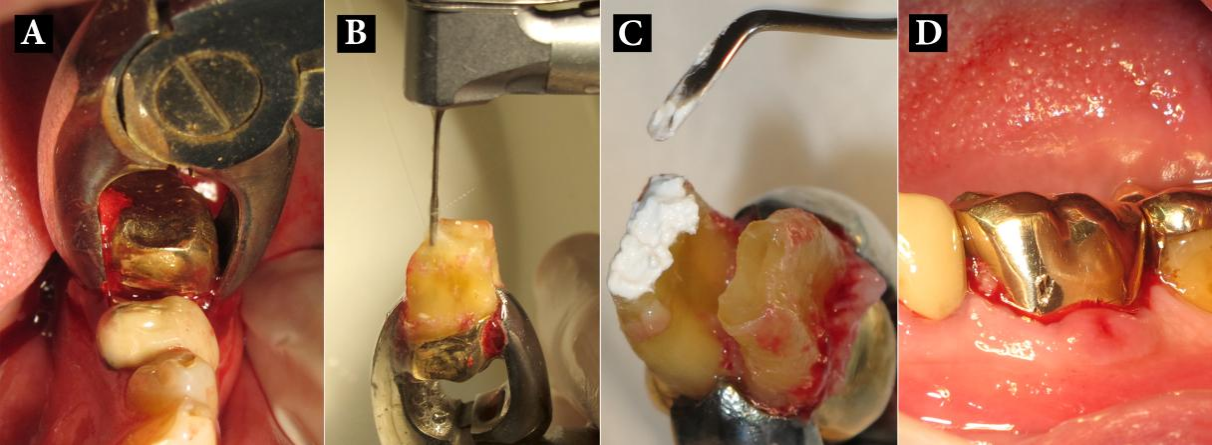
*A)* Pretreatment figure; *B)* Gentle extraction of LR6 with forceps; *C)* Root-end cavity preparation with constant saline irrigation to keep tissue hydrated; *D)* Reinsertion of tooth into socket following extraoral root-end surgery

Extra-oral time was kept minimum (≤15 min). The 2-3 mm root-end resections were performed using diamond bur (Diatech Dental, Coltène-Whaledent, Altstätten, Switzerland); then ~3 mm root-end cavities were prepared using size 3 Gates-Glidden drills (Dentsply, Maillefer, Ballaigues, Switzerland). The root-end cavities were filled with CEM cement (BioniqueDent, Tehran, Iran). Blood clot was aspirated from the extraction socket without curettage. The teeth were then gently replanted into their socket (Figure 1); the accurate repositioning was confirmed by radiography. The teeth did not require stabilization with splints as the teeth were slightly cut-off from occlusion. Antibiotics were not prescribed in any of the cases.

The patients were given postoperative oral hygiene instructions and were asked to use an antiseptic mouthwash (0.2% Chlorhexidine). They were also advised to have a soft diet, and not to chew on the surgery site. The teeth were inspected 1, 7 and 14 days postoperatively via routine intraoral examinations. Follow-ups were planned for +6 months.

IR was deemed successful if radiographic and clinical evidence were supportive. Symptoms of discomfort, tenderness to palpation or percussion were considered as clinical failures. Signs of infection/inflammation (*e.g.* sinus tract, swelling or a deep periodontal pocket) were also regarded as failure. The radiographic outcome was considered as failure if the periapical lesion did not change or it increased in size.

**Table 1:** Summary of IR case studies from 1999

**Year [Ref]**	**Tooth and lesion type**		**n**	**Type of IR treatment**	**Follow-up period ** **(month)**	**Success rates % (** ***n*** **)**
**2011 [**5**]**	Vertical root fracture of anterior teeth	3		IR and adhesion with bonding agent	24	100 (3/3)
**2011 [**10**]**	Severe periodontally involved teeth	12		Enamel matrix derivative and demineralized freeze-dried bone allograft therapy	12	100 (12/12)
**2010 [**18**]**	UR1 Crown root fracture in 10 year old	1		IR 180 degrees rotation followed by RCT and Composite restoration	24	100 (1/1)
**2010 [**19**]**	UL2, UR2, perio-endo lesion due to developmental anomaly	2		Extraoral RCT, flowable composite.	6	100 (2/2)
**2010 [**17**]**	LL6 Furcal perforation	1		Perforation repair and root-end filling with CEM cement	24	100 (1/1)
**2009 [**2**]**	UR2 developmental radicular groove. Periradicular radiolucency	1		Intraoral RCT, IR with emdogain	12 and 48	100 (1/1)
**2008 [**13**]**	UR2 developmental anomaly	1		Extraoral root-end surgery and orthodontic treatment	72	100 (1/1)
**2007 [**6**]**	Maxillary sinusitis related to failed endodontic treatment of UL6	1		Drainage, alveolar curettage and extraoral root-end resection	24	100 (1/1)
**2006 [**20**]**	LL1 periodontal involved low prognosis tooth	1		RCT, extraoral root planning and application of platelet rich plasma	18	100 (1/1)
**2006 [**20**]**	LL6 failed endodontic treatment	1		Intraoral/ extraoral endodontic (re-) treatment and IR	168	100 (1/1)
**2006 [**21**]**	Upper anteriors; trauma related ankylosis	15		Extraoral retrograde titanium, and emdogain on root and in socket	24-72	47 (7/15)
**2004 [**8**]**	Various mainly endodontic problems	9		Extraoral apical surgery	6-192	89 (8/9)
**2004 [**22**]**	Various severity of vertical tooth fractures	26		Extraoral dentin bonding and subsequent metallic post and crown.	4-72	69 (18/26)
**2003 [**7**]**	Anterior tooth crown root fracture	1		RCT and 180º tooth rotation	36	100 (1)
**2003 [**11**]**	LR7 Recurrent endodontic abscess and facial swelling	1		RCT, re-RCT, IR with amalgam, and periodontal pack. Antibiotics were prescribed	180	100 (1/1)
**2002 [**9**]**	UR5 root fracture with mobility and pain and UL5 root fracture	2		180 rotation, bonding root and fixing with orthodontic wire	36	100 (2/2)
**1999 [**23**]**	LR7 endodontic failure	2		Extraoral apical surgery	65	100 (2/2)
**1999 [**1**]**	Teeth requiring retreatment	29		Extraoral apical surgery	12	72 (21/29)

*U; upper, L; lower, R; right, L; left*

## Results

In the present case series, patients opted for IR for various reasons including fear of surgery, clinician recommendation, financial limitations, or strong desire to retain their tooth. Out of the 20 patients undergoing IR, there were 11 females and 9 males with an average age of 37 years. Careful case selection was made by an experienced operator, and then the teeth chosen for IR where atraumatically extracted. Extraoral treatment time was kept within 8-14 min (mean=11.7 min) (Table 2). The preoperative radiographic size of periapical lesions varied between 5 to 10 mm in diameter. There were no pre/postoperative complications such as fracture, traumatic extraction or socket bone fracture during extraction. The patient who used to take warfarin had an INR of 2 and had no complications. 

**Table 2:** Baseline and outcome measures of 20 IR cases

**Case No**	**Tooth**	**Outcome**	**Extraoral time (h)**	**Sex**	**Age**	**Follow-up (m)**
**1**	LR6	success	14	M	25	23
**2**	LL4	success	10	M	45	30
**3**	LR7	success	8	M	41	24
**4**	LR6	success	12	M	23	15
**5**	LR7	success	8	F	46	27
**6**	LR7	success	9	F	31	12
**7**	UR4	failure	10	F	30	18
**8**	LL6	success	13	F	36	14
**9**	LR7	success	14	M	48	16
**10**	LR6	success	14	F	24	8
**11**	UL6	success	14	F	43	17
**12**	LL4	success	12	M	34	15
**13**	LL6	success	10	F	29	11
**14**	LL6	success	14	M	63	12
**15**	UR7	success	13	M	31	10
**16**	LR6	success	14	F	46	8
**17**	LR6	questionable	12	F	40	8
**18**	LR7	success	13	F	27	20
**19**	LL6	success	10	F	41	12
**20**	LR7	success	10	M	37	9
**Mean**	**-**	**90% success**	**11.7**	**-**	**37**	**15.45**

*U; upper, L; lower, R; right, L; left*

**Figure 2:**
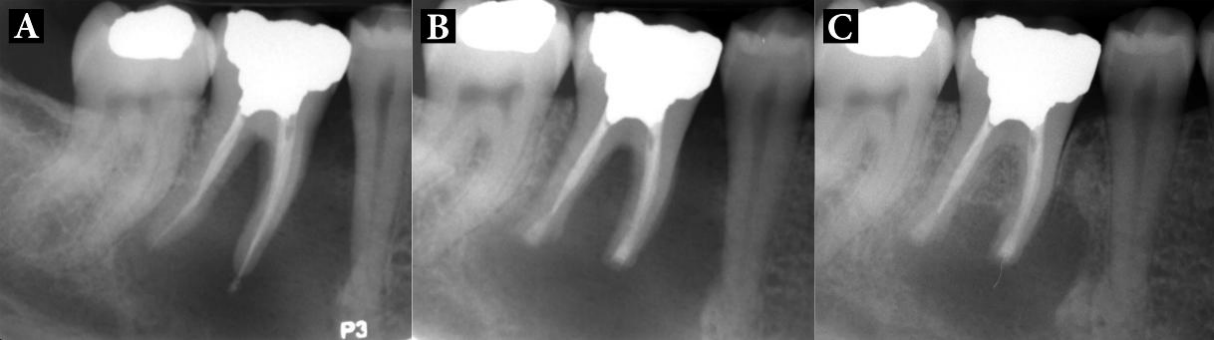
Patient 17: *A)* Preoperative radiograph showing an extensive periradicular lesion; *B)* Immediate post surgical radiograph showing root-end resection/preparation/filling with CEM cement; *C)* As radiographic lesion is incompletely resolved after 8 months, the case is classified questionable

**Figure 3:**
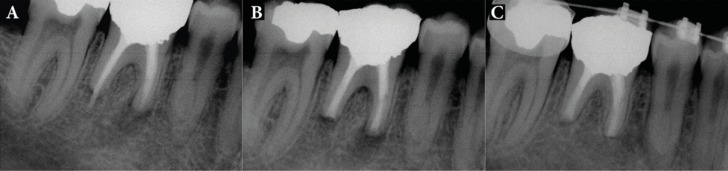
Patient 4: *A)* Patient was referred from orthodontist for treatment of LR6. There is evidence of external root resorption, periapical radiolucency and extension of obturant material. Distal root has an obvious open apex; *B)* IR with extraoral periradicular surgery with CEM cement was performed; *C)* Fifteen-month follow-up radiograph showed complete periodontal regeneration, so that the orthodontic treatment was planned

**Figure 4:**
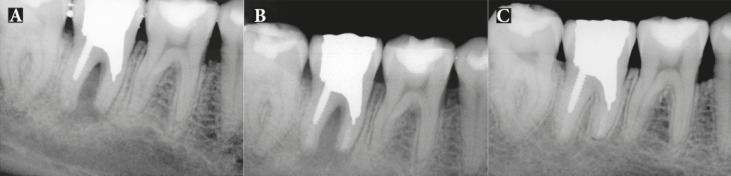
Patient 18: *A)* Patient presented with large periradicular radiolucency and a localized abscess; there was no evidence of root canal treatment; *B)* IR and periradicular endodontic surgery with CEM cement was performed; *C)* Radiograph shows complete resolution of periradicular lesion and normal lamina dura at 20-month follow-up

**Figure 5:**
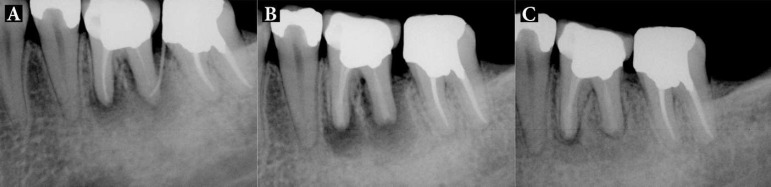
Patient 13: *A)* Periradicular radiolucencies and interdental bone loss; gutta-percha placed into purulent sinus tract; *B)* IR and extraoral periradicular surgery with CEM cement was performed; *C)* Eleven months after IR treatment with periradicular bony infill

**Figure 6:**
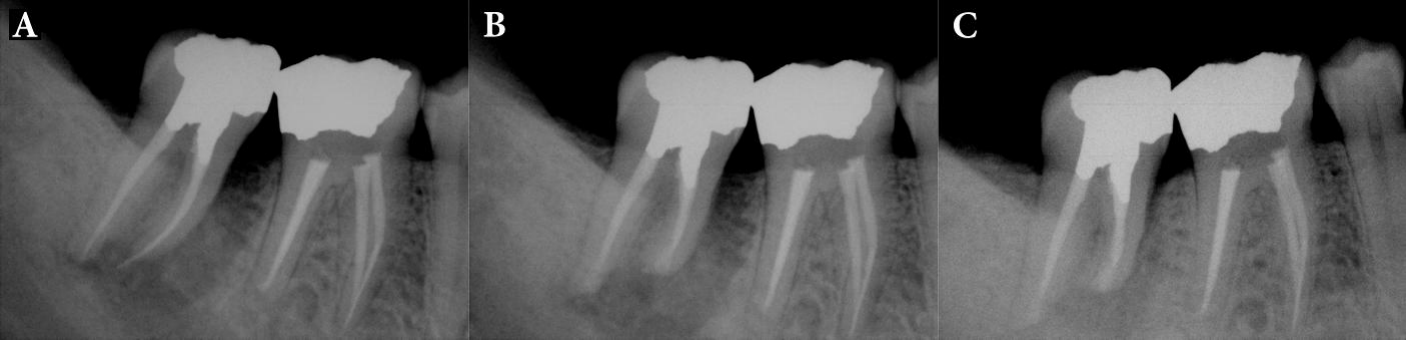
Patient 5: *A)* A wraparound radiolucency at mesial root mimicking vertical root fracture was evident; *B)* IR and extraoral periradicular surgery with CEM cement was performed; *C)* Twenty seven months after IR treatment bone healing is evident

**Figure 7:**
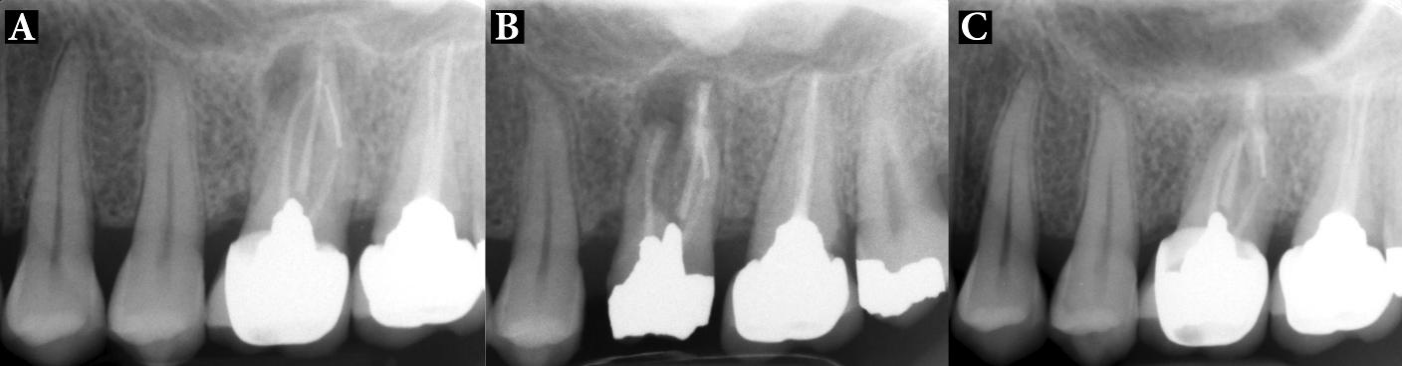
atient 11: *A)* Patient presented with large periradicular radiolucency around the apex of the fused roots of upper first molar ; the root canal treatment is poor and a rather long segment of broken instrument in the distal root is evident; *B)* Immediate post operative radiography; the crown was removed, then IR and periradicular endodontic surgery with CEM cement was done. The prosthetic crown was replaced after three weeks; *C)* Seventeen-month follow-up radiograph shows resolution of periradicular lesion and formation of a normal lamina dura surrounding the roots

**Figure 8:**
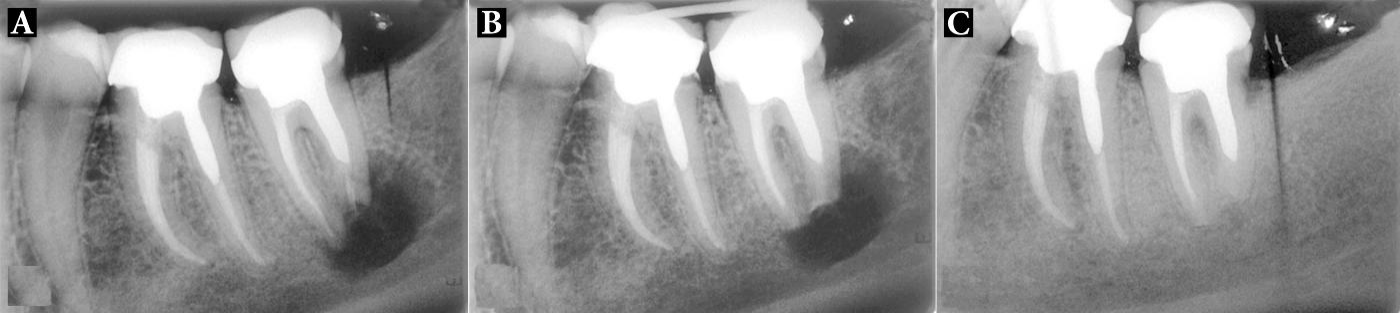
Patient 20: *A)* Poor endodontic treatment resulted in periradicular lesion with extensive post in distal canal; *B)* IR and extraoral periradicular surgery with CEM cement was performed; *C)* Nine month postoperative radiograph; there is complete resolution of the lesion

**Figure 9:**
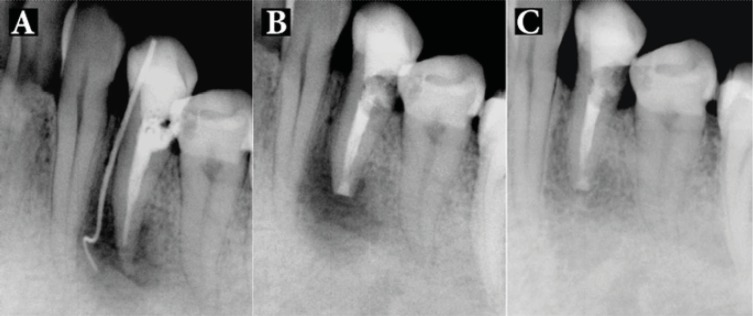
Patient 12: *A)* A premolar with distal perforation, poor endodontic treatment, periradicular radiolucency, purulent sinus tract and poor prognosis; *B)* IR with CEM cement both in apical and at distal perforation regions; *C)* Fifteen-month radiography shows complete resolution of the lesion

Patients were followed-up for 24 months, with the mean being 15.5 months. Total of 18 patients had successful clinical and radiographic outcomes, one upper premolar had failed and one lower molar had questionable prognosis as the radiographic lesion had reduced in size, but not resolved after the 8-month follow-up (Figure 2). All 18 successful cases showed complete resolution of radiographic lesion (Figures 3-9).

## Discussion

Clinical comfort, absence of symptoms, return to function and radiographic resolution are all factors that indicate favourable treatment outcome for IR. Our results show 90% radiographic success rate for IR and extraoral endodontic root-end surgery with CEM cement. Factors that encourage healing, include reduction in extraoral time, atraumatic extraction/reinsertion, prevention of damage to tooth roots, adequate apical seal in terms of depth, material compaction and characteristics as well as suitable case selection. The first two features prevent dehydration and damage to periodontal ligament cells which are essential in the periradicular healing process and prevention of resorptive processes such as replacement resorption, ankylosis, internal and external root resorptions [14]. Traumatically extracted teeth are not good candidates for intentional replantation and that is the reason for careful case selection for IR so that their extraction and subsequent reinsertion would be straight forward (preventing damage to the buccal/lingual plates of the alveolar bone).

There are some absolute contraindications for example, in immune-compromised/suppressed patients, teeth with potential high risk of fracture/trauma (divergent rooted molars), poor patient compliance and oral hygiene. Periodontally involved teeth or fractured teeth may have lower prognosis with IR though the research for an excellent biomaterial is on-going for these cases [24].

Our cases demonstrated that unlike other studies, antibiotics are not needed even when large radiolucent areas are visible radiographically, perhaps due to using a good biomaterial with antimicrobial effects [4, 10, 18]. 

Apart from infection, resorption is also a key reason why IR and traumatically avulsed teeth fail. Missing PDL or necrotic cementum due to excessive extra-oral time, dehydration and/or trauma, may cause replacement resorption or ankylosis. In our cases the IR process spanned between 8-14 min. Most studies, except one [1], revealed the relation between increased extraoral time and resorption [3, 12]. In a published IR case series with the largest amount of cases, the extraoral time ranged between 12-22 min. IR failures were generally due to resorption and/or ankylosis [10]. Compared to most traumatically avulsed teeth, in IR cases there was minimal delay in the reinsertion of the tooth. Therefore, the survival time of IR seems to be longer than avulsed teeth. 

In this case series, IR was deemed suitable for a variety of reasons including operator confidence, intraoral access, tooth coronal restriction, adequate orthograde root treatment (failure possibly due to apical deltas and ramifications), and difficulty of access for periapical surgery. Some patients also had financial restrictions and/or did not want extractions and wished to retain the tooth. 

Our success rate is somewhat higher than other case series *e.g. *the one by Koenig *et al.* with 82% success rate [18], and one other recent case series that reported a 72% success rate [1]. Peer achieved a success rate of 89%, similar to our study [8]. The one case that did fail in that study was deemed as *poor case selection* due to previous widespread periodontal problems. The author challenged the negative view of landmark studies [19] and like our case series he demonstrated high success rate for teeth with root treatment or extra-alveolar apical surgery and IR within 5-10 min [8]. Many more recent case reports have shown 100% success rates for IR performed for a variety of reasons [5, 22, 25]. Most failures occurred due to some form of resorption or periodontal problem [1] which is generally diagnosed after 1

year, however, inflammatory resorption and replacement resorption (ankylosis) can be usually observed after 1-2 months [21]. Clinical trials are required to provide higher level of evidence. 

Comparing success rates of IR is problematic as case selection is critical; moreover the variability in tooth type and follow-up time creates confusion. The various reasons for opting for IR and the etiology of tooth infection/failure, as well as the various extraoral treatments available, greatly influence the prognosis. For instance, root-fractured molars [26], periodontally involved [8] or traumatized teeth with evidence of ankylosis and/or replacement resorption [27] are likely to have lower prognosis compared with endodontically failed teeth.

In our study, the failure of one of the cases (a premolar tooth) was likely to be due to the patient’s medical history. The patient had later developed Systemic Lupus and was on high dose of corticosteroids which may have interfered with the healing process. Also the case with questionable outcome had also significant reduction in lesion size on the radiography and therefore can be termed as “healing”; however it was termed questionable, as the furcal radiolucency had not still resolved after 8 months. 

Other causes for failure of IR can be inadequate root-end filling material and its depth, as well as root resection. The material must have good sealability as this will greatly influence the prognosis of apical endodontic surgery by preventing the penetration or growth of bacteria as the single most common cause of endodontic failures [16]. The role of coronal and apical seal, its effectiveness in preventing re-infection and the ability of biomaterials to induce healing undoubtedly increases the success rate of IR. 

Unlike most previous studies with IR, we used a bioregenerative material with comparable properties to MTA [1, 8]. Several properties are necessary when choosing a root-end filling material including sealing ability, antibacterial activity, and more importantly, cementogenesis; CEM cement is reported to induce cementogenesis as a root-end filling and furcation perforation repair material [17]. It is thought that subsequent to periradicular surgery, mesenchymal cells initiate the healing process by differentiating into mature cells such as osteoblasts, fibroblasts, or cementoblasts thus inducing osseous regeneration and apical attachment healing [16, 17]. The favorable treatment outcomes for CEM cement in this study can be due to its good sealing ability [15], antibacterial activity, high alkalinity [16], hydroxyapatite formation [28], low cytotoxicity [29], biocompatibility [30], and induction of hard tissue formation [31, 32]. Biomaterials may help to make IR a more standard form of therapy, in the right hands. If the long term prognosis of this treatment proves to be high, it may rival success rates of other treatment modalities and possibly be offered as routine treatment. 

The advantageous of IR for the patient include reduction in clinical time, complications and expense compared to non/surgical endodontic (re)treatment. Furthermore, with good case selection, the skilled general practitioner may find IR simpler to perform than endodontic (re)treatment or periradicular surgery. The greatest advantage is the spectacular view and the control that the clinician has to all aspects of the tooth, challenging the last resort treatment argument [33].

## Conclusion

With careful case selection and suitable training, IR can have a high success-rate with bioregenerative material and be far less expensive than other treatment options. Further long term follow-ups will be provided.
